# Development of an image processing software for quantification of histological calcification staining images

**DOI:** 10.1371/journal.pone.0286626

**Published:** 2023-10-05

**Authors:** Xinrui Li, Yau Tsz Chan, Yangzi Jiang

**Affiliations:** 1 School of Medicine, Northwest University, Xi’an, Shaanxi, China; 2 Faculty of Medicine, School of Biomedical Sciences, The Chinese University of Hong Kong, Shatin, Hong Kong SAR, China; 3 Faculty of Medicine, Institute for Tissue Engineering and Regenerative Medicine, School of Biomedical Sciences, The Chinese University of Hong Kong, Shatin, Hong Kong SAR, China; 4 Key Laboratory for Regenerative Medicine, Ministry of Education, School of Biomedical Sciences, The Chinese University of Hong Kong, Shatin, Hong Kong, China; 5 Center for Neuromusculoskeletal Restorative Medicine, Hong Kong Science Park, Shatin, New Territories, Hong Kong, China; 6 Faculty of Medicine, Department of Orthopaedics & Traumatology, The Chinese University of Hong Kong, and Prince of Wales Hospital, Shatin, Hong Kong SAR, China; CESi Engineering School: Ecole d’Ingenieurs CESi, FRANCE

## Abstract

Quantification of the histological staining images gives important insights in biomedical research. In wet lab, it is common to have some stains off the target to become unwanted noisy stains during the generation of histological staining images. The current tools designed for quantification of histological staining images do not consider such situations; instead, the stained region is identified based on assumptions that the background is pure and clean. The goal of this study is to develop a light software named Staining Quantification (SQ) tool which could handle the image quantification job with features for removing a large amount of unwanted stains blended or overlaid with Region of Interest (ROI) in complex scenarios. The core algorithm was based on the method of higher order statistics transformation, and local density filtering. Compared with two state-of-art thresholding methods (*i*.*e*. Otsu’s method and Triclass thresholding method), the SQ tool outperformed in situations such as (1) images with weak positive signals and experimental caused dirty stains; (2) images with experimental counterstaining by multiple colors; (3) complicated histological structure of target tissues. The algorithm was developed in R4.0.2 with over a thousand in-house histological images containing Alizarin Red (AR) and Von Kossa (VK) staining, and was validated using external images. For the measurements of area and intensity in total and stained region, the average mean of difference in percentage between SQ and ImageJ were all less than 0.05. Using this as a criterion of successful image recognition, the success rate for all measurements in AR, VK and external validation batch were above 0.8. The test of Pearson’s coefficient, difference between SQ and ImageJ, and difference of proportions between SQ and ImageJ were all significant at level of 0.05. Our results indicated that the SQ tool is well established for automatic histological staining image quantification.

## Introduction

Histological staining is a method used for labeling and identifying tissues and cells in biomedical research and diagnosis. Histological staining of biological calcification and mineralization relates to skeletal tissue development [1, 2] and tooth formation [3], meanwhile histological staining of pathological calcification in heart valves and arteries could be an early sign of heart diseases [4, 5]. Alizarin Red (AR) and Von Kossa (VK) are two commonly used histological staining methods for the use of identifying calcium components deposited in tissues and cells. Both AR and VK staining methods are widely used in testing osteoblast differentiation, osteocyte and its histopathology-physiological calcification changes in the field of orthopedics and dentistry studies. They are also used for identifying pathological calcification in cardiovascular diseases and other related fields. Therefore, an accurate quantification of the calcified region in AR and VK staining assays at cell and tissue levels is valuable for biological experimental research, disease diagnosis and drug development.

Image recognition is widely applied in quantification of staining areas in the histological staining images [6–10], in which most of these algorithms were developed for recognizing the calcified region based on the object pattern rather than the degree of its color intensity. To the best of our knowledge, there is no methods or tools specifically designed for solving the staining quantification work with massive noisy stains, which is however a common issue in research labs. Images are usually preprocessed by cleaning up its major dirty background manually prior to the automatic quantification phase. An automated tool to quantify the staining results from images is lacked. Therefore, the goal of this study is to develop a tool that could complete the quantification work without manually preprocessing, meanwhile, report results in a batch processing manner.

As of today, the most well-used and widely accepted tool for analyzing histological staining images is ImageJ [10–12], developed by the National Institutes of Health (NIH, USA). This light software of ImageJ is a quick starter for researchers to have the Region of Interest (ROI) outlined by hand-drawing or thresholding, and then report the quantitative outputs accordingly. The use of ImageJ is a general good practice in most laboratories; however, some limitations would hinder its use to reflect the subtle differences at cellular and tissue levels. Firstly, to achieve the best results, most images request to be preprocessed and adjusted manually image-by-image prior to the ROI recognition step, and hence it prevents the use of the software to execute image quantification in a batch processing manner. Secondly, it lacks stability to standardize the outlining and thresholding of the ROI so that the reported results could be biased upon human’s observation. Moreover, since the color and texture can be conterminous between the background noise and the ROI, it is difficult for an automatic recognition to distinguish the background noise from ROI. Current solution for maintaining measurement accuracy for images with massive noises is to define the ROI manually, which is adopted by many software [13] including a commercially available staining image analysis software TissueQuest (TissueGnostics GmbH, Austria).

Behind those wrapped up software, Otsu’s method and Triclass thresholding method are two state-of-art thresholding algorithms for automatic image segmentation. Otsu’s method searches one threshold that segments the image into two classes by minimizing the intra-class variances. Triclass thresholding method separates the image into three classes by the same idea (*i*.*e*., minimizing the intra-class variances) and iteratively execute the segmentation until the class in the middle of foreground and background is completely segmented into the upper and lower classes [14, 15]. Triclass thresholding can be a better choice over Otsu’s method in the case that one or more strong signals exhibited in the image [15]. However, since both Otsu’s method and Triclass thresholding method are solely based on pixel values, they could be unable to distinguish the foreground if its background noise has equal or greater pixel values, or the background noise is overlaid with ROI.

**Fig 1:**
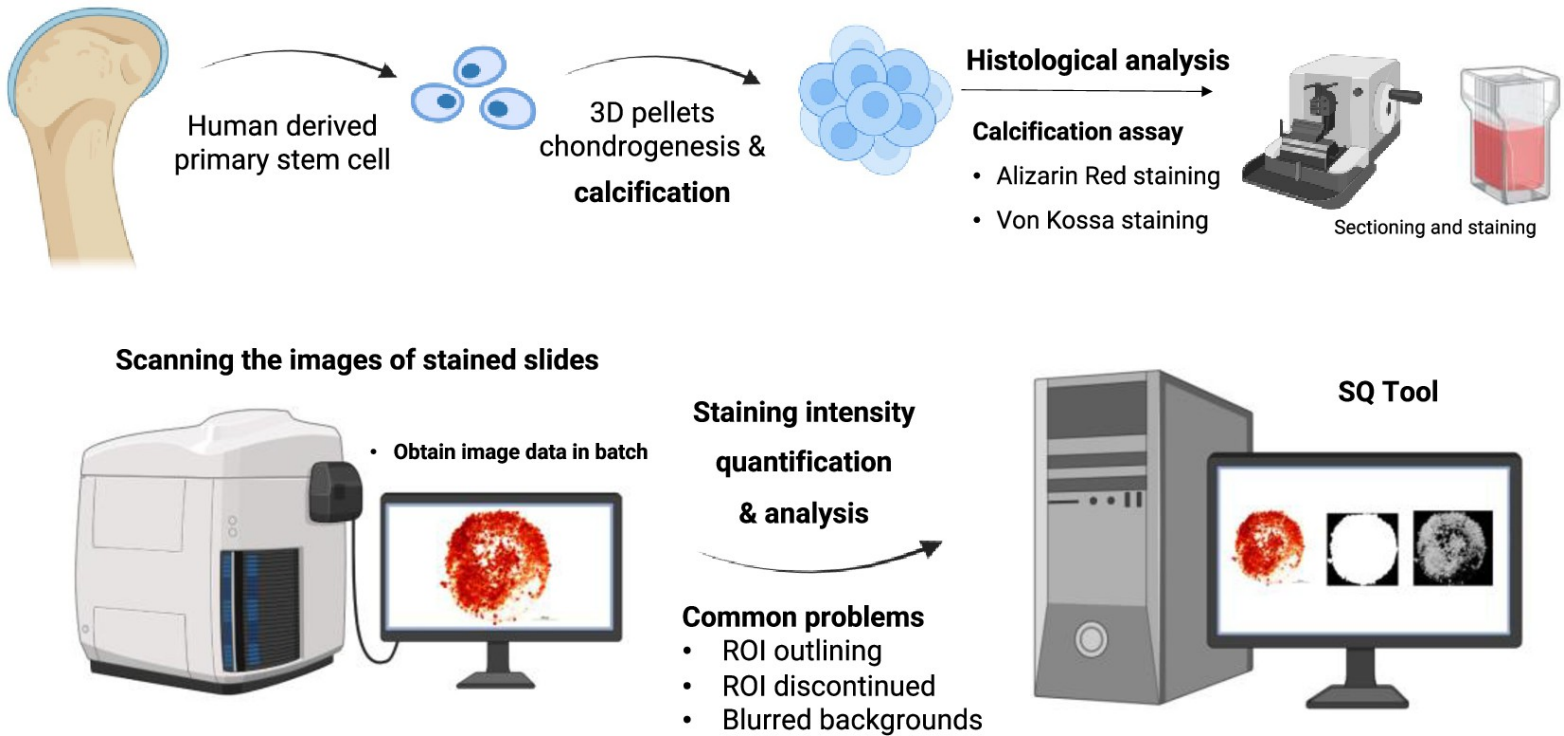
Schematic diagram of the research question and design. Schematic diagram summarized the entire schematic diagram of this study from data collection to image quantification *via* a set of experimental efforts and computational process. The cell sample was originally collected from human derived primary stem cells, and was transferred to 3D pellets for chondrogenesis and calcification. Next, the calcification assay was assigned by applying two types of staining methods and the staining images were photographed for automatic quantification afterwards. Finally, the quantitative readouts along with visualized results were displayed by using the designated SQ tool for staining quantification. This diagram is created with BioRender.com.

Here, we report an unsupervised algorithm for automatic recognition and quantification of histological and cytological staining, especially for calcium staining and quantification (**Fig 1**).

## Materials and methods

### Histological mineralization image acquisition

We obtained more than a thousand histological images of AR and VK staining from our well-established stem cell pellet culture and osteogenic induction model [16–18] (**Fig 1**, top panel). In brief, 2 × 10^5^ human cartilage tissue specific stem/progenitor cells were isolated and cultured into pellets follow our previously protocol [16–18], and using human derived cells as study material was approved by The Chinese University of Hong Kong, the Joint Chinese University of Hong Kong–New Territories East Cluster Clinical Research Ethics Committee (The Joint CUHK-NTEC CREC, Ref. No. 2019.078) with written informed consent obtained. Cells were spun down in V-shaped bottom 96-well plates and cultured for 7 days in high glucose DMEM (Gibco, Waltham, Massachusetts, USA) containing 1× ITS (Gibco), 1× antibiotic-antimycotic (Gibco), 10 ng/ml TGF-β3 (PeproTech, Rocky Hill, NJ, USA), 50 μg/ml ascorbic acid (Sigma, St Louis, MO, USA), 40 μg/ml proline (Sigma), and 0.1 μM dexamethasone (Sigma). The medium was changed every 3–4 days. For induction of mineralization, the cultured pellets were treated with osteogenic medium consisting of high-glucose DMEM (Gibco), 10% FBS (Gibco), 1 × antibiotic-antimycotic (Gibco), 10 mM β-glycerolphosphate (Sigma), 0.1 μM dexamethasone (Sigma), and 50 μg/ml sodium ascorbate (Sigma) for 14 days with medium changed every 3–4 days. These 3D-cultured pellets were then harvested and fixed in 4% paraformaldehyde overnight, followed by tissue processing, paraffin embedding, and sectioning. Five μm-thick sections of the pellets were stained with 2% AR solution (Beyotime, Shanghai, China) at pH 4.2 for 1 minute for AR staining, or with 1% silver nitrate solution (Sigma) for 1 hour under light and counterstained with nuclear fast red for VK staining. The stained sections were dehydrated, mounted, and photographed with Nikon Ni-U Eclipse Upright Microscope (Nikon, Tokyo, Japan).

For external validation (EV) of the algorithm, a set of 50 histological images from previously published papers were selected for representative scenarios of calcification assays [1, 2, 4, 5, 19–36], which included but not limited to developmental of skeletal tissues in developmental biology study [1, 2], calcification in 3D cultured of immortalized mouse myoblast C2C12 cell pellets [19], and calcified blood vessels [4, 5].

### Development of staining quantification (SQ) tool for image quantification

#### HOS transformation

The Higher Order Statistics (HOS) method conceptualized by *Park*, *Jungwoo et al*. [37], was primarily aimed at conducting image segmentation for image sequences with low depth-of-field (DOF). In statistics, the first two moments can derive the mean and variance which depict the average value of a distribution and how spread out the distribution is. The third and fourth moments indicate the asymmetry of a distribution and how heavy its tails or extreme values are [38]. The HOS method calculates the sample mean of the fourth-order central moment at pixel point (*x*, *y*) with a set of its neighboring pixels in a grayscale image. Using the concept of fourth moments, it is possible to transform the original pixel values to their higher order statistics so that a focused object could be segmented from defocused or noisy background. The HOS transformation could be written as:

$${\hat{m}}^{\left(4\right)}(x,y)=\frac{1}{N_{\eta }}\sum_{\left(s,t)\in \eta (x,y\right)}{\left(I(s,t)-\hat{m}(x,y)\right)}^{4}$$

where *η*(*x*, *y*) is a set of neighboring pixels centering at (*x*, *y*), $\hat{m}(x,y)$ is the sample mean of *I*(*s*, *t*).

Since the range of HOS values could be extremely large, the value is down scaled to limit its range to [0, 255]. Therefore, the HOS value can be further written as:

$$HOS(x,y)=min\;(255,{\hat{m}}_{R}^{\left(4\right)}(x,y)/300)$$

where 300 is an appropriate down scaling factor [37].

Unlike the traditional unsupervised image segmentation algorithms, which usually find optimal thresholding value solely based on original pixel values and/or its variance, the HOS method exploits pixels with each pixel surrounded and calculates its kurtosis to identify focused objects by how heavy the kurtosis of a point with its surrounding points could be rather than the pixel value itself.

#### Local density filtering

After the HOS transformation, the original pixel values would be transformed to binary indicators in which focused area tend to have densely distributed points whereas in the defocused or noisy area, the points would be sparsely distributed. This gave a chance for density peaks clustering (DPC) to be able to filter most of the sparse points (*i*.*e*. noisy background) [39–41]. The main idea of DPC is to compute local density for each point and its distance to points of higher density. For each point *p*_*i*_, *i* = 1,…,*N*, there are *k* Nearest Neighbor (KNN) points of *p*_*i*_, which we denote it as *q*_*j*_, *j* = 1,…*k*. Then the average distance of *p*_*i*_ to its nearest neighbors can be defined as:

$$\bar{d_{i}}=1/k∙\sum_{j=1}^{k}d_{ij}$$

where *d*_*ij*_ is the Euclidean distance between *p*_*i*_ and *q*_*j*_. Thus, the local density (LD) can be defined as:

$$LD(p_{i})=1/k∙\sum_{q_{j}\in KNN(p_{i})}exp(\frac{-d_{ij}}{\overset{-}{d_{i}}})$$

*LD*(*p*_*i*_) indicates the magnitude of local density: the lower value of *LD*(*p*_*i*_), the higher possibility that it should be filtered [42].

#### Denoising

With the application of local density filter, ROI would be most likely to be outlined in a rough manner that some very little noise might still leave there. To eliminate those minor objects (noise), we ordered connected objects in sequence according to their size of area, and then eliminate objects with least size of area. The proportion of objects to be deleted was based on a threshold set up by users (normally it is set to be 95%, alike to the confidence interval for a level of significant statistical testing).

#### Positive staining identification

Positive staining region was identified by setting up an appropriate threshold within ROI. Specifically, the positive staining thresholding value for AR was set at 100 (in the range of 0–255) and the positive staining thresholding value for VK was set at 140 (in the range of 0–255).

#### Staining quantification with ImageJ

The histological stained images were also processed and quantified manually with ImageJ (NIH, USA) by two observers independently (XL and YTC). After importing a microscopic photo into ImageJ, the total area of the section was selected either by the “Wand Tool” or by hand drawing if the Wand Tool failed to correctly identify the section. The total area of ROI was then quantified by the “Measure” function of ImageJ and the output was the pixel values. After converting the microscopic photo to 8-bit grayscale image and inverting the image intensity, the positively stained area and intensity were quantified using the “Threshold” function. The positive staining threshold values for Alizarin Red staining was set as 100 (out of 0–255) and the positive staining threshold values for von Kossa staining was set as 140 (out of 0–255). The positive staining intensity percentage was then calculated with the same formula as SQ tool [43]:

$$positive\;staining\;intensity\;\%=\frac{\sum \;intensity\;of\;positively\;stained\;region}{\sum \;maximum\;intensity\;of\;ROI}\times 100\%$$

$$=\frac{\sum \;intensity\;of\;positively\;stained\;region}{area\;of\;ROI\times 255}\times 100\%$$

#### Metrics evaluation and statistical analysis

The main quantitative results from the software included (1) total area, (2) total intensity (in ROI), (3) stained area, and (4) stained intensity (the staining signal within ROI). The comparison was conducted between SQ tool and ImageJ. The accuracy of SQ tool was evaluated by comparing the above four quantitative measurements of SQ tool with ImageJ. AR, VK, and EV images were evaluated separately with a batch of 50 samples each.

Setting ImageJ as a benchmark, the metrics used for evaluating the accuracy were:

- Pearson’s correlation coefficient:

$$r=\frac{N\sum xy-\sum x\sum y}{\sqrt{\left[N\sum x^{2}-{\left(\sum x\right)}^{2}][N\sum y^{2}-{\left(\sum y\right)}^{2}\right]}}$$

where *N* = 50;

x=asetofquantitativeresultsmeasuredbySQtool;

y=asetofquantitativeresultsmeasuredbyImageJ.

- Difference in percentage:

$$diff_{pct}=\frac{x-y}{y}\times 100\%$$

- Difference of proportion in percentage:

$$prop.diff_{pct}=\frac{x_{prop}-y_{prop}}{y_{prop}}\times 100\%$$

where $x_{prop}=\frac{x_{stained}}{x_{total}};\;y_{prop}=\frac{y_{stained}}{y_{total}}$

The 95% confidence interval of the average value for difference in percentage and difference of proportion in percentage was calculated for each batch, and t test was conducted at a significance level of 0.05. The null hypothesis was: the absolute value of true mean is greater than 0.05.

- Success rate:

A difference in percentage and a difference of proportion in percentage less than 0.05 will be considered as a success image recognition, and the success rate will be calculated for each batch accordingly.


$$success\;rate=\frac{N(diff_{pct}(or\;prop.diff_{pct})<0.05)}{N}$$

## Results

### Overall workflow of SQ tool

**Fig 2:**
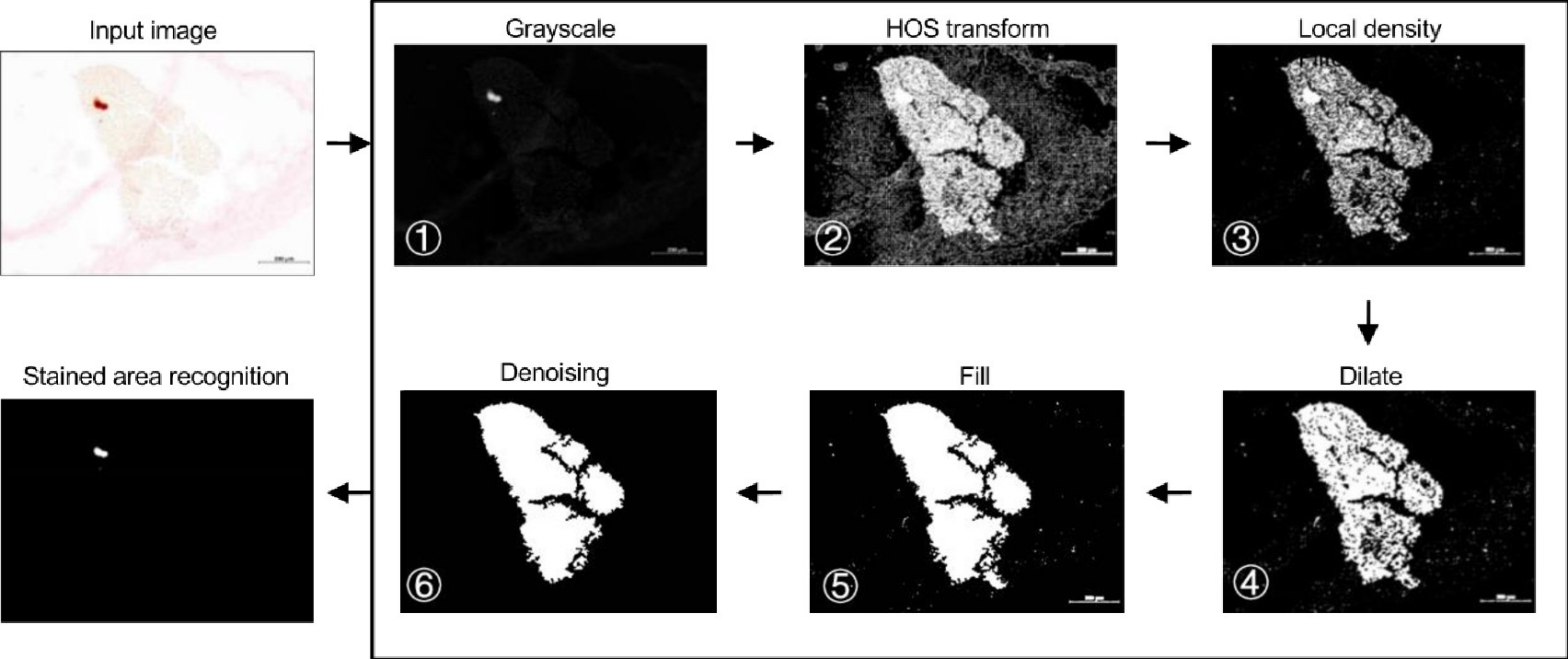
Workflow of total tissue and stained area segmentation. Input image is a histological staining image of Alizarin Red Stating of stem cell pellet underwent osteogenic differentiation. The study subject in this image is stained in light yellow; the positive staining area is in orange red, and the cloudy light pink zones are noise signals (bar = 200 μm). The automatic staining quantification (SQ) algorithm includes six steps successively, in which three major steps (HOS transform, local density filter, denoising) were conducted to check with different types of noises.

The methodology of SQ tool includes three main phases: Higher Order Statistics (HOS) transformation, local density filtering, and denoising. **Fig 2** shows the complete workflow of image processing and quantification, including six steps: 1) grayscale; 2) HOS transformation; 3) local density filtering 4) dilate 5) fill in solid 6) denoising. The details of the main methodologies are as below.

### Comparison of HOS transformation with conventional methods

**Fig 3:**
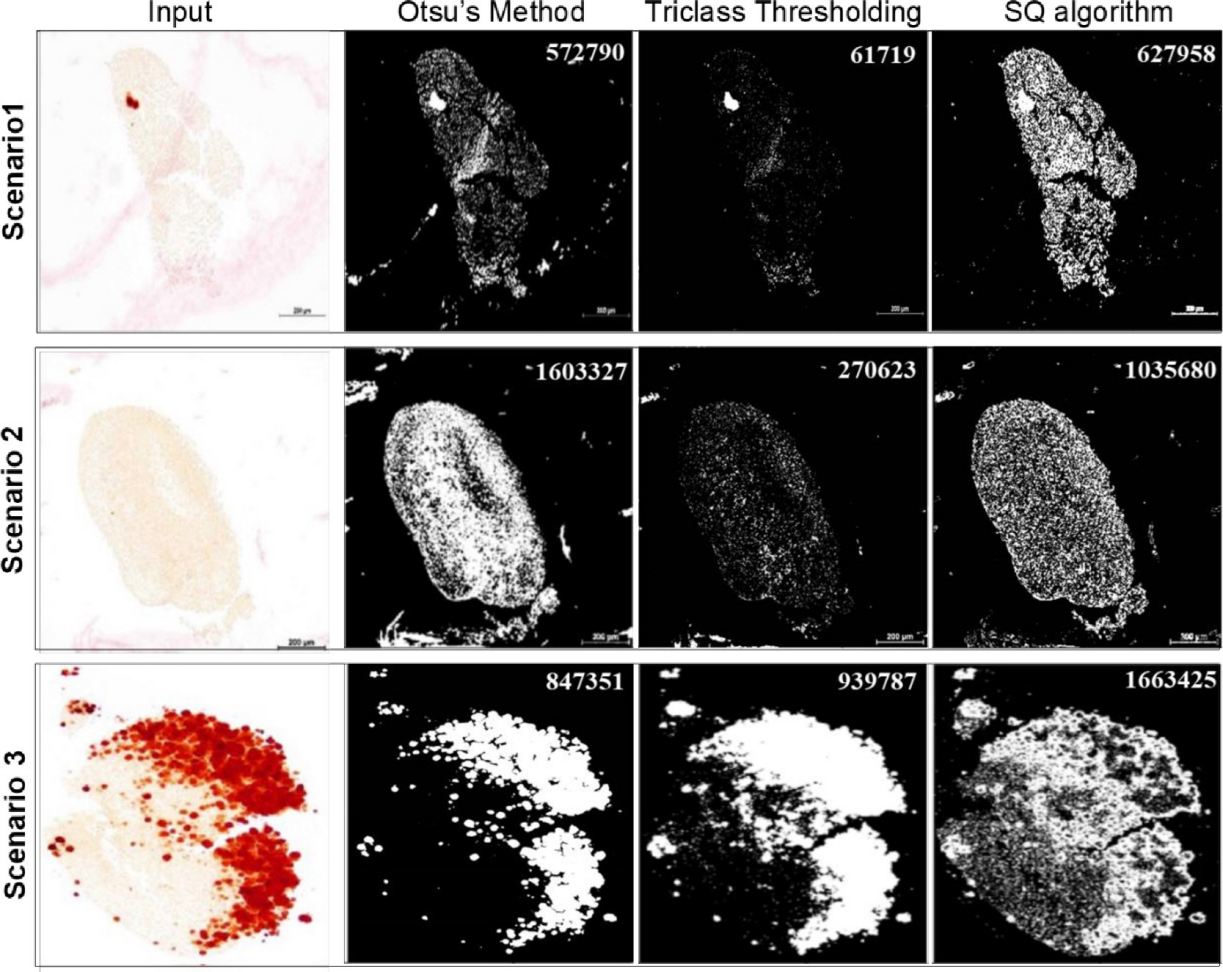
Comparison of HOS transformation with Otsu’s method and Triclass thresholding. A comparison of HOS transformation with two traditional segmentation methods. HOS transformation outperformed the other two methods in three different scenarios which stand for most representative problems in histological images of Alizarin Red Stating from stem cell pellet underwent osteogenic differentiation, bar = 200 μm. Pixel values of the identified region were displayed in each graph. **Scenario 1** shows that when the image contains dirty background and tiny positive stained signal, in the meantime, the dirty background might have stronger intensity than the ROI, the former two methods were unable to clear dirty background or outline the ROI exclusively. **Scenario 2** shows that when there is no positive stained signal and the dirty background was completely blended with the ROI, the overlapping area cannot be distinguished or the method failed to outline the ROI. SQ algorithm successfully segmented the blending dirt and gave clear ROI outline. **Scenario 3** shows that when the image contains massive positive stained signal, the former two methods would usually miss some part of ROI (e.g., some weak signals) since the color contrast is too strong.

**Fig 3** shows the results from three scenarios recognized by HOS method compared with two traditional methods (Otsu’s method and Triclass thresholding method) [14, 15]. The binary indicators in **Fig 3** indicates the primary interested area with white (labeled as 1) and not-interested area with black (labeled as 0). HOS method has superior performance than the above-mentioned methods in identifying the tissue area in all three scenarios. In **Fig 3** scenario 1, in the case that positive signal is weak, the blurry dirt is all over the background and ROI and its intensity is stronger than ROI, Otsu’s method fails to exclude the blurry dirt (pixel value is 572,790) and Triclass thresholding fails to include most part of ROI (pixel value is 61,719). On the other hand, HOS method identifies the ROI without blurry dirt (pixel value is 627,958). In **Fig 3** scenario 2, in the case that there is no positive signal and the blurry dirt is connected or overlaid with ROI, Otsu’s method is unable to distinguish between the connected blurry dirt and ROI (pixel value is 1,603,327) and Triclass thresholding fails to include most part of ROI (pixel value is 270,623). On the other hand, HOS method separates the connected blurry dirt from ROI and have the ROI identified correctly (pixel value is 1,035,680). In **Fig 3** scenario 3, in the case that the positive signal is strong and massive compared to the rest part of ROI, although Triclass thresholding was reported to be superior if one or more strong signals are exhibited in the image [15], in current practice Otsu’s method and Triclass thresholding fails to identify most part of the ROI (pixel value is 847,351 for Otsu’s method, 939,787 for Triclass method). In contrast, HOS method correctly identifies ROI with the weak intensified area included (pixel value is 1,663,425). More details of ROI are reserved by HOS method because it transformed pixel values to the sample mean of the fourth-order central moment at pixel point (*x*, *y*) with a set of its neighboring pixels.

### Data readouts from SQ tool in recognizing of Alizarin Red staining and von Kossa staining

**Fig 4:**
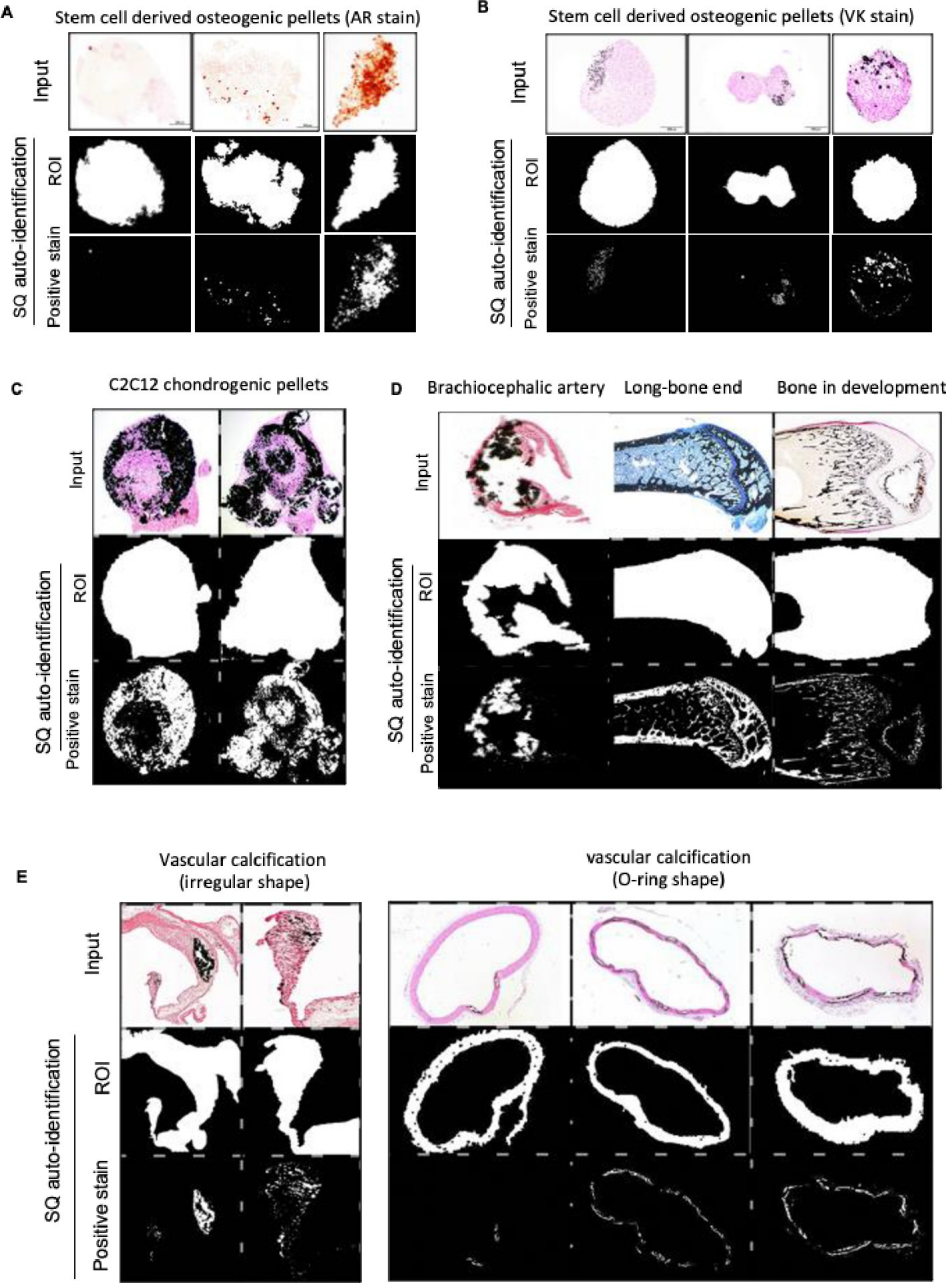
Reprehensive images ROI and positive stain recognition of SQ tool of histological calcification images. (**A**) Reprehensive images of stem cell pellets underwent osteogenic differentiation with Alizarin Red, and (**B**) Reprehensive images of stem cell pellets Von Kossa staining (bar = 200 μm). Original images can be found in **S2A Fig** (n = 50 for AR) and **S2B Fig** (n = 50 for VK). (**C-E**) External Validation calcification-stained images: to validate the ROI auto-recognition function of SQ algorithm, we collected histological images from published papers about mineralization of chondrogenic pellets (**C**), brachiocephalic artery study, bone biology study, bone developmental biology study (**D**), and vascular calcification study (**E**). These images are all stained with von kossa to identify tissue calcification, and counterstained with hematoxylin erosion (H&E), or von Kossa/McNeal’s tetrachrome staining. Original images of EV images (n = 50) are listed in **S2C Fig**, with permissions of reprint.

With above method established, the main outputs of SQ tool at subject recognition step included: two black and white pictures indicating tissue area (ROI) and staining area recognized (as the representative images in **Fig 4**), and the descriptive statistics such as intensity, area, and percentage of the staining area.

To validate the SQ tool, we examined 50 images of AR staining (**Fig 4A**, **S1A Fig**), and another 50 images of VK staining (**Fig 4B**, **S1B Fig**) of the stem cell pellet cultures generated in our lab. Besides in-house data, the accuracy of readouts of SQ tool was further verified with external published images represent the most common calcification scenarios in biomedical research. A total of 50 random images including Alizarin Red and von Kossa staining were collected from published papers [1, 2, 4, 5, 19–36] (**Fig 4C–4E**, and **S1C Fig**).

### Comparison of the readouts from SQ tool with ImageJ

**Fig 5:**
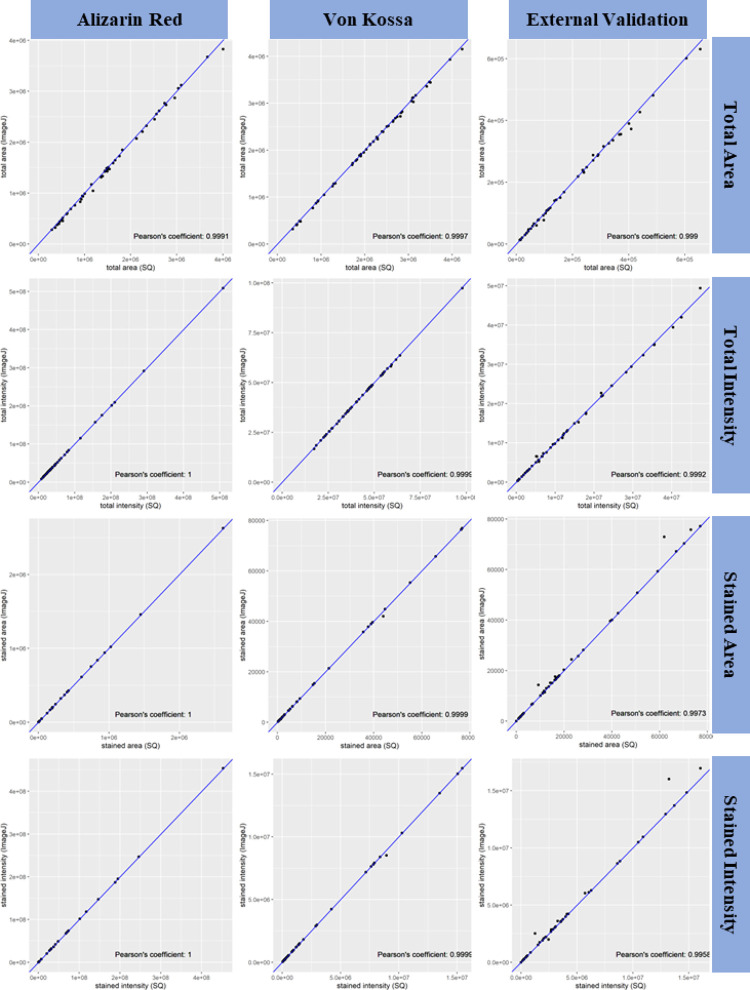
Pearson’s coefficient between SQ and ImageJ. Pearson’s coefficient between SQ (x axis) and ImageJ (y axis) measured for the metrics of total area, total intensity, stained area, and stained intensity in the three batches of 50 samples for Alizarin Red, Von Kossa, and external validation, respectively. The blue line is the diagonal line with slope = 1. Pearson’s coefficient is shown in the lower right of each graph.

**Fig 6:**
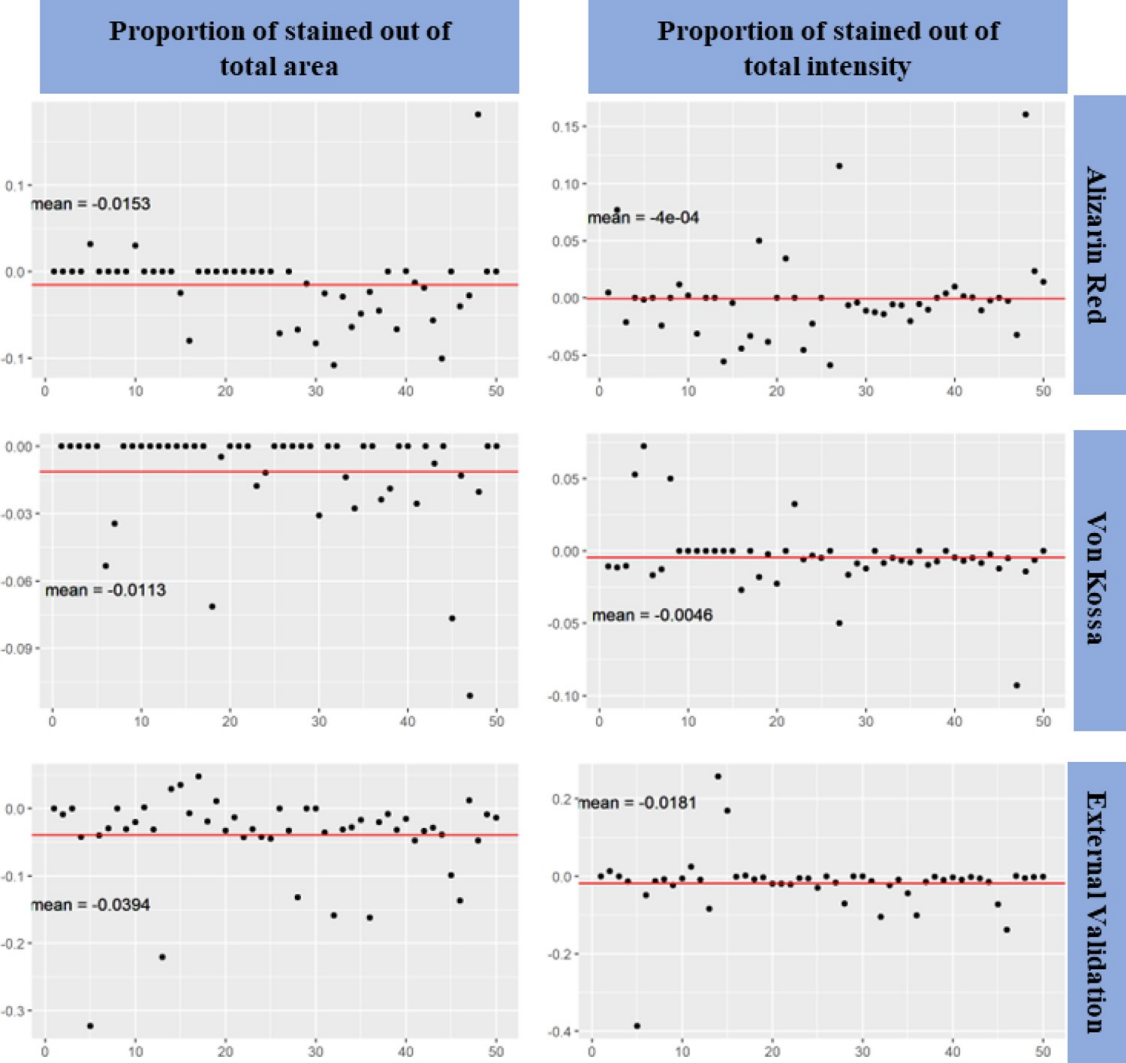
Difference of proportion between SQ and ImageJ. Difference of proportions between SQ and ImageJ measured for the metrics of stained area out of total area, and stained intensity out of total intensity in the three batches of 50 samples for Alizarin Red, Von Kossa, and external validation, respectively were shown in each graph. The red line is the average of difference of proportion in percentage and their values were shown correspondingly.

We then compared the results obtained from SQ tool and ImageJ, and discussed for the metrics of Pearson’s correlation coefficient, difference in percentage between SQ and ImageJ, difference of proportion in percentage between SQ and ImageJ for the batch of AR, VK, and EV set, respectively (**Figs 5** and **6**). **Table 1** shows the complete comparison of SQ and ImageJ in multi-levels. The difference in percentage and difference of proportion in percentage was measured by its sample mean, 95% confidence interval, p value of t test with null hypothesis of true mean being greater than 0.05, and success rate.

**Table 1:** Evaluation metrics of SQ tool compared with ImageJ in multi-levels.

Metrics—level1	Metrics—level2	batch	value	95% CI (%)	p value
Pearson’s coefficient	total area	Alizarin Red	0.9993	-	<0.0001
		Von Kossa	0.9997	-	<0.0001
		External Validation	0.9990	-	<0.0001
	total intensity	Alizarin Red	1.0000	-	<0.0001
		Von Kossa	0.9999	-	<0.0001
		External Validation	0.9992	-	<0.0001
	stained area	Alizarin Red	1.0000	-	<0.0001
		Von Kossa	0.9999	-	<0.0001
		External Validation	0.9973	-	<0.0001
	stained intensity	Alizarin Red	1.0000	-	<0.0001
		Von Kossa	0.9999	-	<0.0001
		External Validation	0.9958	-	<0.0001
Difference in percentage (%)	total area	Alizarin Red	2.48	(1.60, 3.36)	<0.0001
		Von Kossa	1.47	(1.02, 1.92)	<0.0001
		External Validation	2.67	(1.16, 4.18)	0.0020
	total intensity	Alizarin Red	0.38	(0.03, 0.72)	<0.0001
		Von Kossa	0.53	(0.36, 0.69)	<0.0001
		External Validation	-0.04	(-1.46, 1.37)	<0.0001
	stained area	Alizarin Red	0.21	(-0.62, 1.05)	<0.0001
		Von Kossa	0.01	(-0.63, 0.65)	<0.0001
		External Validation	-1.68	(-3.23, -0.14)	<0.0001
	stained intensity	Alizarin Red	0.37	(-0.52, 1.26)	<0.0001
		Von Kossa	0.13	(-0.48, 0.74)	<0.0001
		External Validation	-1.84	(-4.16, 0.48)	0.0052
Proportional difference in percentage (%)	stained area out of total area	Alizarin Red	-1.53	(-2.71, -0.34)	<0.0001
		Von Kossa	-1.13	(-1.76, -0.49)	<0.0001
		External Validation	-3.94	(-5.74, -2.14)	<0.0001
	stained intensity out of total intensity	Alizarin Red	-0.04	(-1.07, 0.99)	<0.0001
		Von Kossa	-0.46	(-1.09, 0.18)	<0.0001
		External Validation	-1.81	(-3.96, 0.34)	<0.0001

#### Pearson’s correlation coefficient

As shown in **Table 1** and **Fig 5**, the Pearson’s coefficient between data obtained from SQ and ImageJ for total area, total intensity, stained area, and stained intensity in the three batches (AR, VK, and EV) were all high. **Fig 5** shows that most points were lying on the diagonal line or around it and **Table 1** shows that the 12 measurements were all near to 1. These results indicate that the quantitative results of SQ and ImageJ are highly correlated with almost a perfect positive relationship. The Pearson’s coefficients provided a preliminary analysis and comparison of the four measurements between SQ and ImageJ and showed that the results obtained by SQ tool were consistent with results obtained by ImageJ.

#### Difference in percentage between SQ and ImageJ

**Table 1** shows minute difference in percentage between SQ and ImageJ for total area, total intensity, stained area, and stained intensity in the three batches (AR, VK, and EV). The absolute value of average sample mean of the 12 quantitative measurements were all less than 0.05 (*ave*. Mean <0.05) and their corresponding p value were all significant (p<0.05). This illustrates that the null hypothesis of true mean being greater than 0.05 can be rejected. Among the 12 measurements, total intensity in the EV batch, stained area in the AR and VK batch, stained intensity in the AR, VK and EV batch have 95% CI for average sample mean lying through zero, which means that those measurements have 95% confidence to conclude that SQ had no difference from ImageJ in terms of above 6 measurements. **S2 Fig** shows the scatterplots of the four quantitative measurements in the three batches. Most points were randomly distributed around zero, with a few outliers.

Setting the difference less than 0.05 as a standard for success, the success rate was also shown in **Table 1**. SQ tool could automatically recognize and evaluate more than 90% of the AR and VK images and more than 80% of the EV images and the results were as good as obtained manually from Image J.

#### Difference of proportion in percentage between SQ and ImageJ

Since the proportion of stained area (stained area/total area) and the proportion of stained intensity (stained intensity/total intensity) are two mostly useful readouts in data quantification, the difference of proportion was calculated in addition to the directly readouts difference. **Table 1** shows the difference of proportion in percentage. Similarly, the absolute value of sample mean of the 6 quantitative measurements were all less than 0.05 (ave. Mean <0.05) and their corresponding p value was <0.0001. We can conclude that there is more than 99.9% confidence that the true mean of difference of proportion in percentage between SQ and ImageJ were less than 0.05 (ave. Mean). The 95% CIs included zero for difference of stained intensity in all three batches (AR, VK, and EV).

**Fig 6** also shows that most points of the difference of proportion were randomly distributed around zero. In terms of the success rate of data processing, SQ tool could automatically evaluate the stained area and staining intensity in more than 80% (**Table 1**) of the images (**S1A–S1C Fig**), and the results were as good as obtained manually from Image J.

**Fig 7:**
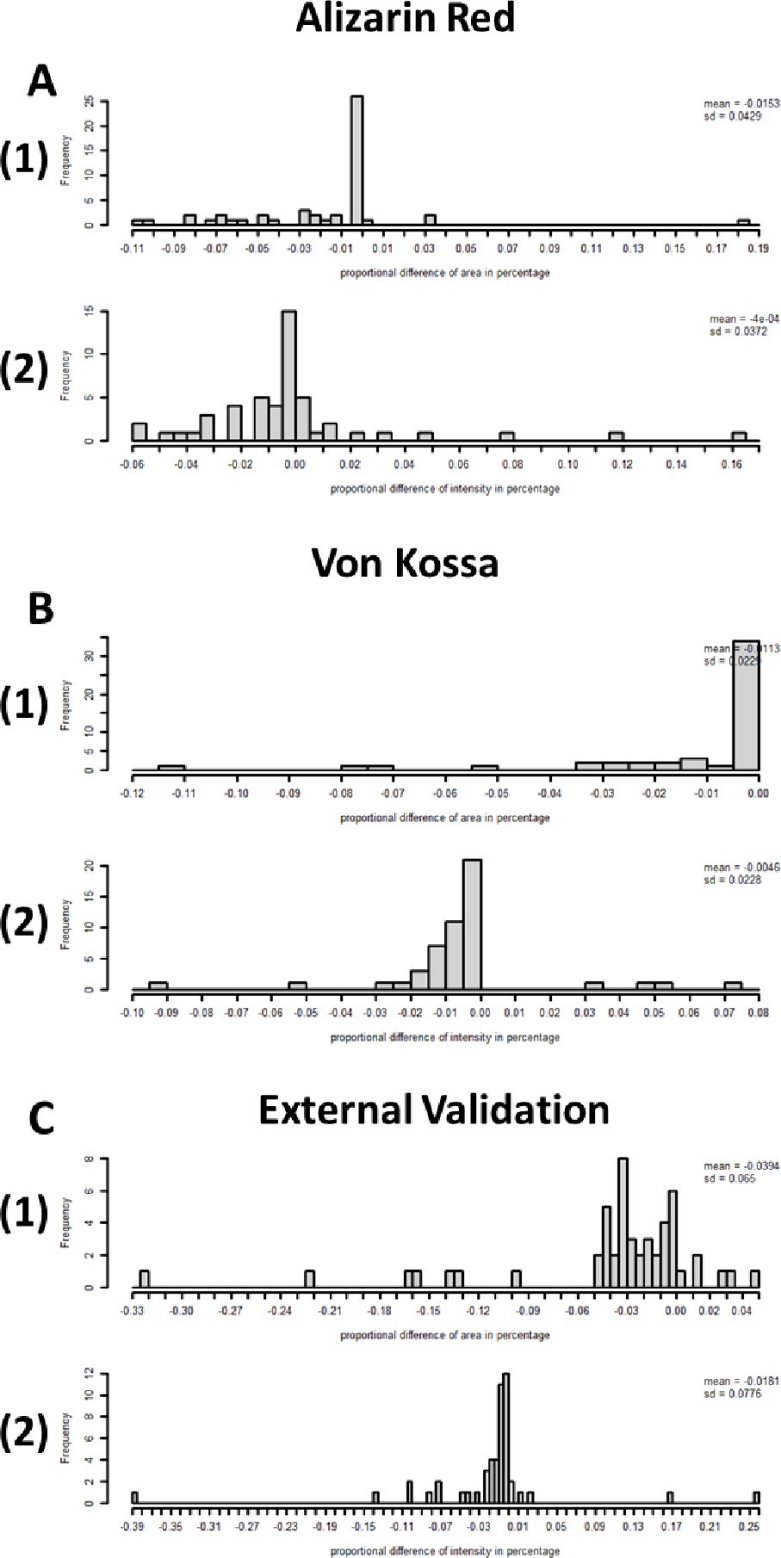
Histogram of proportional difference in percentage. The batch of 50 samples for Alizarin Red(A), Von Kossa(B), and external validation set(C) were arranged vertically to show the histogram of proportional difference in percentage between SQ and ImageJ for the stained area out of total area(1), and the stained intensity out of total intensity(2).

The histogram in **Fig 7** shows the difference of proportion for the measurements of area (**Fig 7A1–7C1**) and intensity (**Fig 7A2–7C2**). The highest frequency values were around zero, with a few observations being very low or high, which suggests manual check is needed for these outliers.

## Discussion

We developed an unsupervised image recognition algorithm incorporated in the SQ Tool that could process images stained in different batches with diverse noisy background, and could standardize the staining intensity and provide reliable quantification results of the common calcification staining images. The SQ tool is highly automatic with the ability of high-throughput batch processing and parameter-tuning. We firstly collected histological images of AR and VK, which were generated from our laboratory to evaluate the degrees of stem cell osteogenesis with a three-dimensional *in vitro* tissue culture model [18, 44], and developed the algorithm by R 4.0.2 (**Fig 1**). The image was taken the step of grayscale at first, and then the background noise was optimized by the steps of HOS transformation, local density filtering, dilate, fill and denoising. We then validated the application of this algorithm in other scenarios with histological images of skeletal tissue development, dental slides, and calcified arteries [1, 2, 4, 5, 19].

To date, the widely used quantification tool for histological staining images was ImageJ. Some of recent research have developed analogous image analysis tools for quantification, but they either acquire basic quantitative measurements from ImageJ to achieve derivative functions [10], or the tools were designed to quantify the input batch images without noisy stains [9]. We then compared the readouts from SQ tool to the readouts obtained from ImageJ in several metrics including results of area and intensity in both stained region and total ROI region. The Pearson’s coefficients were all above 0.99, and the hypothesis tests of difference in percentage (<0.05) were all showed significance. The quantitative evaluations demonstrate that SQ tool can produce results as good as ImageJ. Thus, we concluded that SQ tool is smarter and quicker in quantification of calcification staining signals compared to ImageJ. Given the accuracy as we tested, SQ tool can get similar results as ImageJ and solve the specific quantification problem of histological staining images on tissue or cell with complicated backgrounds.

Compared to ImageJ, the SQ tool is highly automatic and high-throughput, thus saves time and labor cost, and has advantages in the following aspects: (**i**) image preprocessing is time-consuming and could add uncontrolled artificial factors. SQ tool does not require images to be preprocessed to remain only interested regions. Images can be diagnosed directly from experimental outputs. (**ii**) A large amount of images can be diagnosed simultaneously, so that they would be ruled under same thresholding criteria. This will enhance the reliability to the quantification and avoid the observer bias during manual thresholding. (**iii**) The software is semi-parametric, which means that parameters such as binary cut-off value for HOS transformation, degree of dilate, percent cut-off value for denoising are enabled to make adjustment. (**iv**) The most important feature of SQ tool is to automatically recognize noisy stains blended with ROI. It is common to have debris and other noisy signals in the histological staining images obtained from wet lab, and occasionally some stains would be blended with the background and get out of the interested region (e.g., **Fig 3**, Scenario 1; **Fig 4A** top left). Sometimes the unwanted stains (e.g., red and pink stains generated by Eosin) have the same or stronger color and intensity as those calcification staining signals, and they are difficult to distinguish by human’s eyes. The essential difference between noisy stains and the interested stains in ROI is that the noisy stains are defocused during the experimental process of dyeing. Hence, in this study, the concept of HOS transformation is to treat those noisy stains as defocused regions and such that disturbing stains would be transformed to very dispersive points compared to the focused stains in ROI (**Fig 3**). With that being processed, computers can outline ROI in a manner of separation between dispersive points and centralized points.

SQ tool is a lightweight and functional tool with easy application in biomedical research, compared to the supervised machine learning methods such as neural network (NN)-based algorithms, whose labeling and model training can take an enormous amount of time and computation resources. NN-based algorithms would be superior in recognizing certain objects (*e*.*g*. cars, animals, human beings) given the pattern of those objects and it requires a vast amount of images with similar kind of pattern to train the model. On the other hand, the SQ tool created a label-free segmentation that requires light computational resource with fast processing speed.

Despite that the tool has achieved promising results with very high level of accuracy, there are several critical challenges and flaws need to be considered for further development. For instance, the default parameters can be adjusted for a batch of images, but prior check is required for each batch and the parameters would still need to be tuned by observer. Future revision could be to add machine learning algorithm to fine-tune the parameters instead of offering a list of default parameters static for all images.

In conclusion, we developed a lightweight, fast diagnostic image recognition tool specifically aimed at quantification of staining images. The use of it could be extended to other staining image recognitions not limited to AR and VK staining.

## Supporting information

S1 Fig Original images of histological calcification assays. Three batches of samples for (**A**) Alizarin Red (from 50 random samples), (**B**) Von Kossa staining (from 50 random samples) of stem cell cultured pellets. Samples of stem cell pellet underwent osteogenic differentiation with Alizarin Red and Von Kossa staining positive signals exhibited in an ascending order according to the percentage of positive staining area (bar = 200 μm). (**C**) External Validation calcification-stained images (from 22 published papers. Images with permission of reuse were listed, images with no permission of reused regranted were included in the reference [20, 21, 27, 32–34]). To validate the ROI auto-recognition function of SQ algorithm, we collected histological results images and from published papers about mineralization of chondrogenic pellets, brachiocephalic artery study, bone biology study, bone developmental biology study, and vascular calcification study with permissions of reprint. These images are all stained with von kossa to identify the tissue calcification, and counterstained with hematoxylin erosion (H&E), or von Kossa/McNeal’s tetrachrome staining.
(PDF)

S2 Fig Difference in percentage between SQ and ImageJ. Difference in percentage between SQ and ImageJ measured for the metrics of total area, total intensity, stained area, and stained intensity in the batch of 50 samples for Alizarin Red, Von Kossa, and external validation, respectively. The red line is the average of difference in percentage and the value is shown in each graph.
(PDF)

## A list of abbreviations

AR: Alizarin Red
VK: Von Kossa
EV: External Validation
SQ: Staining Quantification
ROI: Region of Interest
HOS: Higher Order Statistics
NN: Neural Network
DPC: Density Peaks Clustering

## References

[1] Ye L., et al., Osteopetrorickets due to Snx10 deficiency in mice results from both failed osteoclast activity and loss of gastric acid-dependent calcium absorption. PLoS genetics, 2015. 11(3): p. e1005057. doi: 10.1371/journal.pgen.1005057 25811986 PMC4374855

[2] Schneider M.R., Von Kossa and his staining technique. Histochemistry and Cell Biology, 2021: p. 1–4. doi: 10.1007/s00418-021-02051-3 34799748 PMC8695535

[3] Baranova J., et al., Tooth formation: are the hardest tissues of human body hard to regenerate? International journal of molecular sciences, 2020. 21(11): p. 4031. doi: 10.3390/ijms21114031 32512908 PMC7312198

[4] Borland S.J., et al., X-ray Micro-Computed Tomography: An Emerging Technology to Analyze Vascular Calcification in Animal Models. International Journal of Molecular Sciences, 2020. 21(12): p. 4538. doi: 10.3390/ijms21124538 32630604 PMC7352990

[5] McCabe K.M., et al., Calcitriol accelerates vascular calcification irrespective of vitamin K status in a rat model of chronic kidney disease with hyperphosphatemia and secondary hyperparathyroidism. Journal of Pharmacology and Experimental Therapeutics, 2018. 366(3): p. 433–445. doi: 10.1124/jpet.117.247270 29903718

[6] Rodrigues É.O., et al., A novel approach for the automated segmentation and volume quantification of cardiac fats on computed tomography. Computer methods and programs in biomedicine, 2016. 123: p. 109–128. doi: 10.1016/j.cmpb.2015.09.017 26474835

[7] Graffy P.M., et al., Automated segmentation and quantification of aortic calcification at abdominal CT: application of a deep learning-based algorithm to a longitudinal screening cohort. Abdominal Radiology, 2019. 44(8): p. 2921–2928. doi: 10.1007/s00261-019-02014-2 30976827

[8] Bortsova G., et al., Automated Segmentation and Volume Measurement of Intracranial Internal Carotid Artery Calcification at Noncontrast CT. Radiology: Artificial Intelligence, 2021. 3(5): p. e200226. doi: 10.1148/ryai.2021200226 34617024 PMC8489463

[9] Guo X., et al., SCU‐Net: A deep learning method for segmentation and quantification of breast arterial calcifications on mammograms. Medical physics, 2021. 48(10): p. 5851–5861. doi: 10.1002/mp.15017 34328661

[10] Eggerschwiler B., et al., Automated digital image quantification of histological staining for the analysis of the trilineage differentiation potential of mesenchymal stem cells. Stem cell research & therapy, 2019. 10(1): p. 1–10. doi: 10.1186/s13287-019-1170-8 30808403 PMC6390603

[11] Rasband, W.S. ImageJ. 1997–2018; Available from: https://imagej.nih.gov/ij/.

[12] Xu L., et al., Digital Image Analysis-Based Evaluation of Claudin-1 and Claudin-7 Delocalization in Cutaneous Squamous Cell Carcinoma and in Its Precancerous State. Journal of Oncology, 2022. 2022: 2750193. doi: 10.1155/2022/2750193 35432533 PMC9007676

[13] Zareian R. and Kheradvar A.,METHOD FOR IDENTIFICATION AND QUANTIFICATION OF TISSUE CALCIFICATION, USPTO, Editor. 2020: United States of America.

[14] Otsu N., A threshold selection method from gray-level histograms. IEEE transactions on systems, man, and cybernetics, 1979. 9(1): p. 62–66.

[15] Cai H., et al., A new iterative triclass thresholding technique in image segmentation. IEEE transactions on image processing, 2014. 23(3): p. 1038–1046. doi: 10.1109/TIP.2014.2298981 24474373

[16] Jiang Y., et al., Human cartilage-derived progenitor cells from committed chondrocytes for efficient cartilage repair and regeneration. Stem Cells Translational Medicine, 2016. 5(6): p. 733–744. doi: 10.5966/sctm.2015-0192 27130221 PMC4878331

[17] Jiang Y., et al., Cartilage stem/progenitor cells are activated in osteoarthritis via interleukin-1β/nerve growth factor signaling. Arthritis research, 2015. 17(1): p. 1–13. 10.1186/s13075-015-0840-x PMC4650403 26577823

[18] Jiang Y. and Tuan R.S., Role of NGF‐TrkA signaling in calcification of articular chondrocytes. The FASEB Journal, 2019. 33(9): p. 10231–10239. doi: 10.1096/fj.201900970 31238006

[19] Li G., et al., The dose of growth factors influences the synergistic effect of vascular endothelial growth factor on bone morphogenetic protein 4–induced ectopic bone formation. Tissue Engineering Part A, 2009. 15(8): p. 2123–2133. doi: 10.1089/ten.tea.2008.0214 19215221 PMC2811054

[20] Balachandran K., et al., Elevated cyclic stretch induces aortic valve calcification in a bone morphogenic protein-dependent manner. The American journal of pathology, 2010. 177(1): p. 49–57. doi: 10.2353/ajpath.2010.090631 20489151 PMC2893650

[21] Bonavita C.M., et al., Characterization of murine cytomegalovirus infection and induction of calcification in Murine Aortic Vascular Smooth Muscle Cells (MOVAS). Journal of Virological Methods, 2021. 297: p. 114270. doi: 10.1016/j.jviromet.2021.114270 34461152 PMC8511269

[22] Choe N., et al., miR-27a-3p targets ATF3 to reduce calcium deposition in vascular smooth muscle cells. Molecular Therapy-Nucleic Acids, 2020. 22: p. 627–639. doi: 10.1016/j.omtn.2020.09.030 33230462 PMC7578555

[23] De Maré A., et al., Sclerostin as regulatory molecule in vascular media calcification and the bone–vascular axis. Toxins, 2019. 11(7): p. 428. doi: 10.3390/toxins11070428 31330917 PMC6669501

[24] Hammers D.W., et al., The D2. mdx mouse as a preclinical model of the skeletal muscle pathology associated with Duchenne muscular dystrophy. Scientific Reports, 2020. 10(1): p. 1–12. 32826942 10.1038/s41598-020-70987-y PMC7442653

[25] Huber A.R., et al., Esophageal mucosal calcinosis: a rare site of gastrointestinal mucosal calcinosis. The American Journal of Case Reports, 2018. 19: p. 406. doi: 10.12659/ajcr.908255 29622763 PMC5900467

[26] Janda K., et al., Vascular effects of advanced glycation end-products: content of immunohistochemically detected AGEs in radial artery samples as a predictor for arterial calcification and cardiovascular risk in asymptomatic patients with chronic kidney disease. Disease Markers, 2015. 2015: 153978. doi: 10.1155/2015/153978 25852219 PMC4380091

[27] Li H., et al., Corneal calcification of acellular porcine corneal stroma following lamellar keratoplasty. Acta Ophthalmologica, 2022. 100(2): p. 164–174. doi: 10.1111/aos.14665 33258298

[28] Moss A.J., et al., Ex vivo 18F-fluoride uptake and hydroxyapatite deposition in human coronary atherosclerosis. Scientific Reports, 2020. 10(1): p. 1–9. 33214599 10.1038/s41598-020-77391-6 PMC7677392

[29] Ruderman I., et al., Vascular calcification in skin and subcutaneous tissue in patients with chronic and end-stage kidney disease. BMC nephrology, 2020. 21(1): p. 1–10. doi: 10.1186/s12882-020-01928-0 32677907 PMC7364566

[30] Song M., et al., Yak pericardium as an alternative biomaterial for Transcatheter Heart Valves. Frontiers in bioengineering, 2021: p. 1076. doi: 10.3389/fbioe.2021.766991 34820366 PMC8607193

[31] Staessens S., et al., Detailed histological analysis of a thrombectomy-resistant ischemic stroke thrombus: a case report. Thrombosis Journal, 2021. 19(1): p. 1–7. 33618719 10.1186/s12959-021-00262-1 PMC7901204

[32] Tani T., et al., Inhibition of tissue‐nonspecific alkaline phosphatase protects against medial arterial calcification and improves survival probability in the CKD‐MBD mouse model. The Journal of pathology, 2020. 250(1): p. 30–41. doi: 10.1002/path.5346 31509234 PMC7238767

[33] Vo T., et al., Injectable dual-gelling cell-laden composite hydrogels for bone tissue engineering. Biomaterials, 2016. 83: p. 1–11. doi: 10.1016/j.biomaterials.2015.12.026 26773659 PMC4754149

[34] Wanga S., et al., Aortic microcalcification is associated with elastin fragmentation in Marfan syndrome. The Journal of pathology, 2017. 243(3): p. 294–306. doi: 10.1002/path.4949 28727149

[35] Zhu L., et al., Hyperhomocysteinemia induces vascular calcification by activating the transcription factor RUNX2 via Krüppel-like factor 4 up-regulation in mice. Journal of Biological Chemistry, 2019. 294(51): p. 19465–19474. 31628194 10.1074/jbc.RA119.009758 PMC6926465

[36] Zhang J.-K., et al., Protection by salidroside against bone loss via inhibition of oxidative stress and bone-resorbing mediators. PloS one, 2013. 8(2): p. e57251. doi: 10.1371/journal.pone.0057251 23437352 PMC3577746

[37] Park J. and Kim C. Extracting focused object from low depth-of-field image sequences. in Visual Communications and Image Processing 2006. 2006. International Society for Optics and Photonics.

[38] Blitzstein J.K. and Hwang J., Introduction to probability. 2015: Crc Press Boca Raton, FL.

[39] Wang R. and Zhu Q., Density peaks clustering based on local minimal spanning tree. IEEE Access, 2019. 7: p. 108438–108446.

[40] Yu D., et al., Density peaks clustering based on weighted local density sequence and nearest neighbor assignment. IEEE Access, 2019. 7: p. 34301–34317.

[41] Celebi M.E., Aslandogan Y.A., and Bergstresser P.R.. Mining biomedical images with density-based clustering. in International conference on information technology: coding and computing (ITCC’05)-volume II. 2005. IEEE.

[42] Ning X., et al., An efficient outlier removal method for scattered point cloud data. PloS one, 2018. 13(8): p. e0201280. doi: 10.1371/journal.pone.0201280 30070995 PMC6072004

[43] Guzman C., et al., ColonyArea: an ImageJ plugin to automatically quantify colony formation in clonogenic assays. PloS one, 2014. 9(3): p. e92444. doi: 10.1371/journal.pone.0092444 24647355 PMC3960247

[44] Jiang Y., et al., Incorporation of bioactive polyvinylpyrrolidone–iodine within bilayered collagen scaffolds enhances the differentiation and subchondral osteogenesis of mesenchymal stem cells. Acta biomaterialia, 2013. 9(9): p. 8089–8098. doi: 10.1016/j.actbio.2013.05.014 23707501

