# Supplementary figures and images for: Development of an image processing software for quantification of histological calcification staining images
Source: PLoS One. 2023 Oct 5;18(10):e0286626. doi: 10.1371/journal.pone.0286626 (PMC10553316; doi:10.1371/journal.pone.0286626)

**A****Alizarin Red**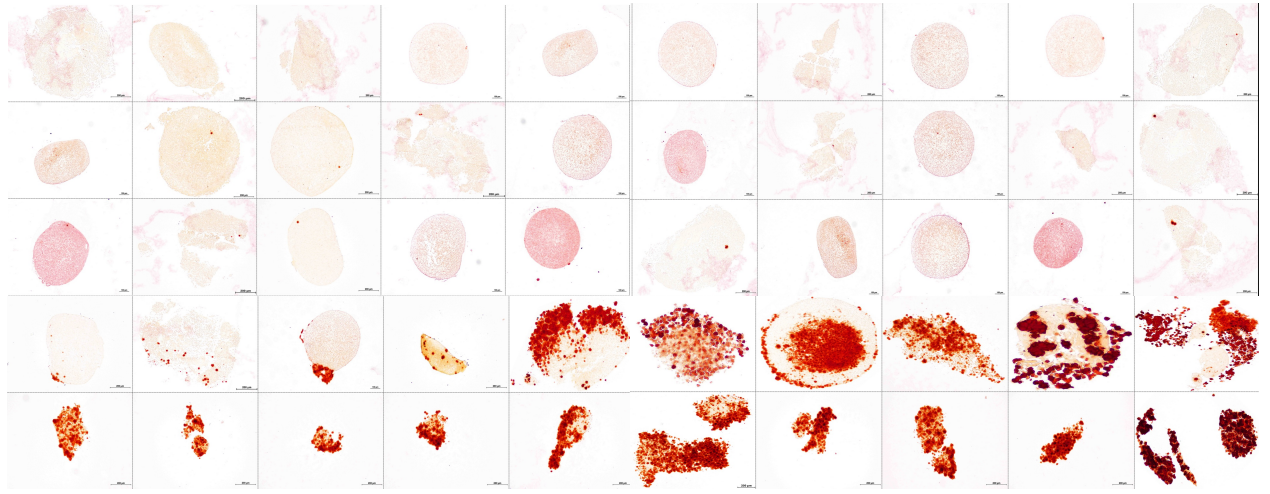**B****Von Kossa**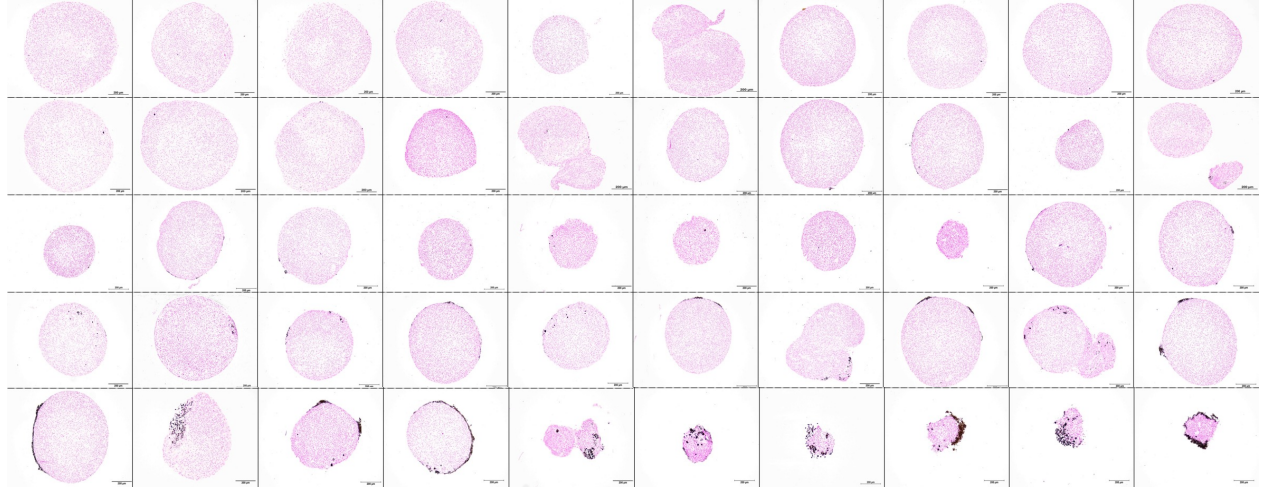**C****External Validation**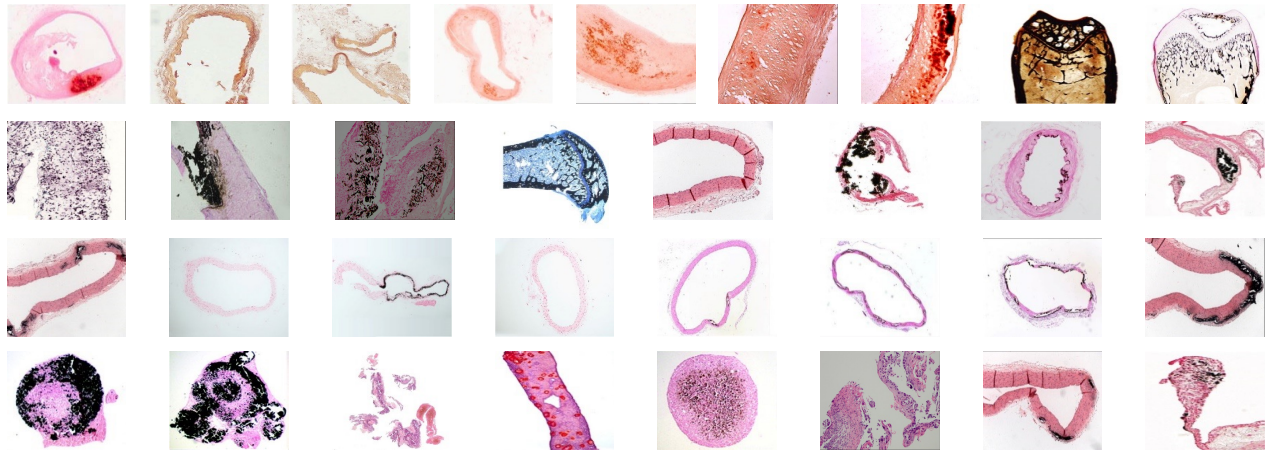

Supplement: S1 Fig — Three batches of samples for (A) Alizarin Red (from 50 random samples), (B) Von Kossa staining (from 50 random samples) of stem cell cultured pellets. Samples of stem cell pellet underwent osteogenic differentiation with Alizarin Red and Von Kossa staining positive signals exhibited in an ascending order according to the percentage of positive staining area (bar = 200 μm). (C) External Validation calcification-stained images (from 22 published papers. Images with permission of reuse were listed, images with no permission of reused regranted were included in the reference [20, 21, 27, 32–34]). To validate the ROI auto-recognition function of SQ algorithm, we collected histological results images and from published papers about mineralization of chondrogenic pellets, brachiocephalic artery study, bone biology study, bone developmental biology study, and vascular calcification study with permissions of reprint. These images are all stained with von kossa to identify the tissue calcification, and counterstained with hematoxylin erosion (H&E), or von Kossa/McNeal’s tetrachrome staining. (PDF) [file pone.0286626.s001.pdf]

# Difference in percentage between SQ and ImageJ

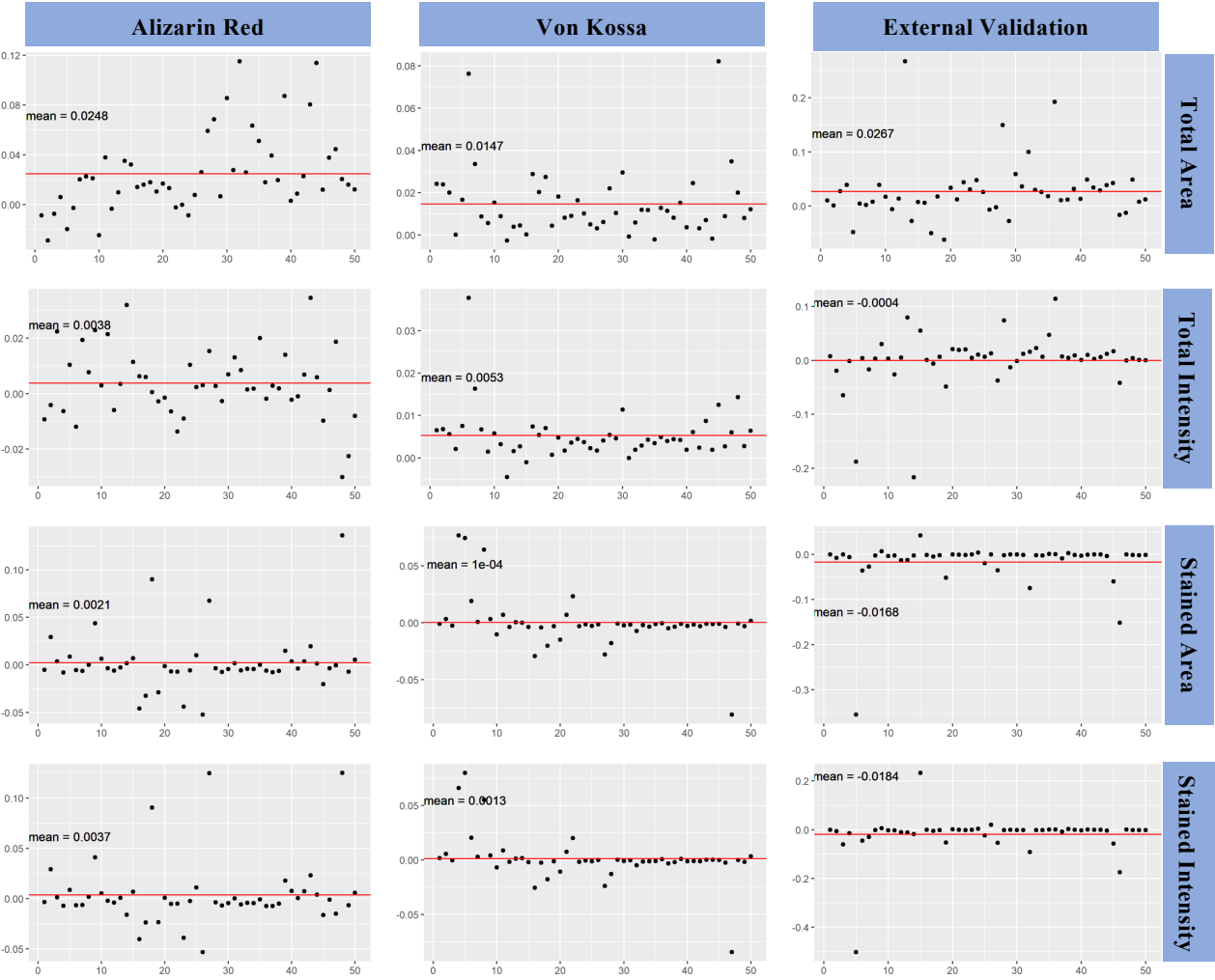

Supplement: S2 Fig — Difference in percentage between SQ and ImageJ measured for the metrics of total area, total intensity, stained area, and stained intensity in the batch of 50 samples for Alizarin Red, Von Kossa, and external validation, respectively. The red line is the average of difference in percentage and the value is shown in each graph. (PDF) [file pone.0286626.s002.pdf]
